# Synthetic Cannabinoid Activity Against Colorectal Cancer Cells

**DOI:** 10.1089/can.2018.0065

**Published:** 2018-12-21

**Authors:** Wesley M. Raup-Konsavage, Megan Johnson, Christopher A. Legare, Gregory S. Yochum, Daniel J. Morgan, Kent E. Vrana

**Affiliations:** ^1^ Department of Pharmacology, Pennsylvania State University College of Medicine, Hershey, Pennsylvania.; ^2^ Department of Biochemistry and Molecular Biology, Pennsylvania State University College of Medicine, Hershey, Pennsylvania.; ^3^ Department of Anesthesiology, Pennsylvania State University College of Medicine, Hershey, Pennsylvania.

**Keywords:** colorectal cancer, synthetic cannabinoids, CBD, THC

## Abstract

**Introduction:** Colorectal cancer (CRC) is a leading cause of cancer-related deaths worldwide, and new therapeutic strategies are still required. Here we screened a synthetic cannabinoid library to identify compounds that uniformly reduce the viability of seven CRC cell lines.

**Material and Methods:** Seven distinct CRC cell lines were treated with 10 μM cannabinoid compounds (from a library of 370 molecules) for 48 h, and cell viability was subsequently measured with MTS assay. Dose–response curves were conducted for compounds that were found to reproducibly reduce cell viability of one or more cell lines.

**Results:** We identified 10 compounds from the library that were able to reduce cell viability of CRC cell lines (with an IC_50_ ≤ 30 μM). Of these compounds, seven were specific for CRC cells, and six were effective in all CRC cell lines tested. Treatment with traditional phytocannabinoids (THC or CBD) was either ineffective or much less potent and only partially efficacious. Treatment with antagonists for the known cannabinoid receptors (alone or in combination) failed to block the activity of the most potent of identified compounds.

**Conclusion:** We identified three families of cannabinoid compounds that reduce CRC cell viability through a noncanonical receptor mechanism. Future modification of these compounds may lead to the development of novel therapies to treat this disease.

## Introduction

With an estimated 97,220 new cases and over 50,000 deaths each year, colorectal cancer (CRC) is the third most common cancer and the third most common cause of cancer death.^1,2^ Mutations in genes comprising the Wnt/β-catenin pathway that leads to an uncontrolled activation of this pathway are found in nearly all colorectal tumors.^3,4^ These mutations contribute to uncontrolled cell proliferation, making this pathway an attractive target for therapeutic development. Despite this knowledge, attempts to target the Wnt/β-catenin pathway to inhibit cancer growth have been largely unsuccessful. In light of these facts, new therapeutic approaches need to be undertaken.

Over the past decade, the number of countries and states that have legalized medical cannabis has grown rapidly, and cannabinoid compounds may serve as a novel therapeutic agent to combat a number of diseases. With regard to cancer, medical cannabis has largely been utilized for palliative purposes^5–7^; however, a number of studies have proposed the use of cannabinoid compounds as anti-tumor agents.^8–11^ These plant-derived cannabinoids interact with the endocannabinoid system and have a much higher affinity for the receptors than do endogenous ligands. The expression of cannabinoid receptors 1 and 2 (CB1 and CB2) and GPR55 has been reported to have increased in CRC, and this is associated with a poorer prognosis and more advanced disease.^12–17^

The objective of our study was to identify compounds from a synthetic cannabinoid library that universally reduces cell viability of seven distinct CRC cell lines (SW480, SW620, HT-29, DLD-1, HCT116, LS174, and RKO). Importantly, these cell lines represent a diversity of tumor types: the first four cell lines contain mutations in *APC*, the most common activating mutation of the Wnt/β-catenin pathway in CRCs; in contrast, HCT116 and LS174 have direct activating mutations in *CTNNB1*, the gene that encodes β-catenin, while RKO cells have intact Wnt/β-catenin signaling. SW480 and SW620 cells were isolated from the same individual, with SW480 cells representing a primary tumor and SW620 a lymph node metastatic tumor. Our approach of analyzing CRC cell lines with distinct Wnt signaling mutations allowed us to determine whether the genetic lesion or tumor subtype could influence sensitivity to cannabinoid treatment. Here we identified several cannabinoid compounds that show potential as novel therapies to combat CRC. Intriguingly, these agents do not appear to act through traditional cannabinoid receptor signaling.

## Methods

### Cell lines

The human CRC cell lines SW480, SW620, HT29, DLD-1, HCT115, LS174, RKO; the temperature-sensitive t-antigen-transformed normal human colonic epithelial cell line CCD 841 CoTr; and the human embryonic kidney cell line HEK 293 were obtained from the American Type Culture Collection (ATCC). Each line, with the exception of the RKO line that was cultured in RPMI, was cultured in Dulbecco's modified Eagle's medium supplemented with 10% fetal bovine serum, 2 mM Glutamax, 10 U/mL penicillin, 10 μg/mL streptomycin, and 0.25 μg/mL Amphotericin B at 37°C in 5% CO_2_. CCD 841 CoTr cells were cultured as described above, but grown at the permissive temperature of 33°C.

### RNA isolation and reverse transcribed-quantitative polymerase chain reaction

RNA isolation and cDNA synthesis were performed as previously described.^18,19^ Target gene expression was measured by quantitative real-time polymerase chain reaction using TaqMan™ probes for *CNR1* (Hs01038522_s1), *CNR2* (Hs00275635_m1), *GPR55* (Hs00271662_s1), *TRPV1* (Hs00218912_m1), and *GAPDH* (Hs99999905_m1) (ThermoFisher; Waltham, MA). Relative levels were determined using the 2^−ΔΔCT^ method.^20^

### Viability screening

Cells were seeded in 96-well plates at a density of 20,000 cells per well, incubated for 8 h, and then treated with members of a synthetic cannabinoid library (Cayman Chemical, Ann Arbor, MI) at 10 μM. This library contains 370 distinct molecules consisting of parent compounds along with positional isomers, analogs, and homologs. Cells were treated with the compounds for 48 h, and then cell viability was measured using the MTS assay following the manufacturer's protocol (Promega, Madison, WI). Cells were incubated with MTS for 1.25 h (2 h for LS174 cells, due to a delay in color development observed with this cell line), after which absorbance at 590 nm was measured using a FlexStation 3 spectrophotometer (Molecular Devices, San Jose, CA). Cell viability for DMSO-treated controls was set to 100%. A z-score was calculated for individual plates, and compounds that displayed a z-score of −1.5 or greater were selected for repeat screening (i.e., compounds that decreased viability by ≥1.5 standard deviations from the mean for the entire screening plate). Importantly, any compounds identified that reduced viability of one of the cell lines tested were rescreened against all cell lines, to reduce the possibility that potential compounds would be overlooked. These experiments were supplemented with the traditional phytocannabinoids (CBD; THC) at the same concentration. For select compounds, MTS results were confirmed with trypan blue staining. Cells were plated and treated as described, and after 48 h, adherent and nonadherent cells were collected, stained with 0.2% trypan blue, and counted on a hemocytometer.

### Dose–effect curves

Any compounds that reduced viability upon rescreening in either the original cell line or in a second cell line were pursued, and dose–response curves were performed on these compounds for all cell lines. Cells were seeded as described above, incubated for 8 h, and treated with select cannabinoid compounds at concentrations of 100 nM, 333 nM, 1 μM, 3.3 μM, 5.6 μM, 10 μM, 33 μM, and 100 μM. Viability was measured as described in the viability screening section.

### Antagonist experiments

To explore the receptors mediating cell death, experiments were conducted using selective antagonists to inhibit each of the four primary cannabinoid receptors. SW480 cells were seeded as described in the viability screening section and incubated for 8 h. The cells were then treated with 10 μM receptor antagonist with or without 5 μM (±)-5-epi CP 55,940. Antagonists used were Rimonabant (CB1-selective), SR 144528 (CB2-selective), ML-193 (GPR-55-selective), and SB-705498 (TRPV1-selective) (Cayman Chemical, Ann Arbor, MI). Following negative preliminary findings, experiments were repeated combining all four antagonists. Viability was measured by MTS assay as described above.

### Statistical analysis

Each compound identified in the primary screen that reduced viability was rescreened two additional times; therefore, each potential compound identified from the original screen was tested three times at 10 μM in all seven cell lines. Moreover, 30 compounds were subjected to replicate (*n*=3) dose–effect curves in all of the cell lines. Statistical significance was determined using the Student's *t*-test (with *p*≤0.05 considered statistically significant).

## Results

### Cannabinoid receptor expression

**FIG. 1.**
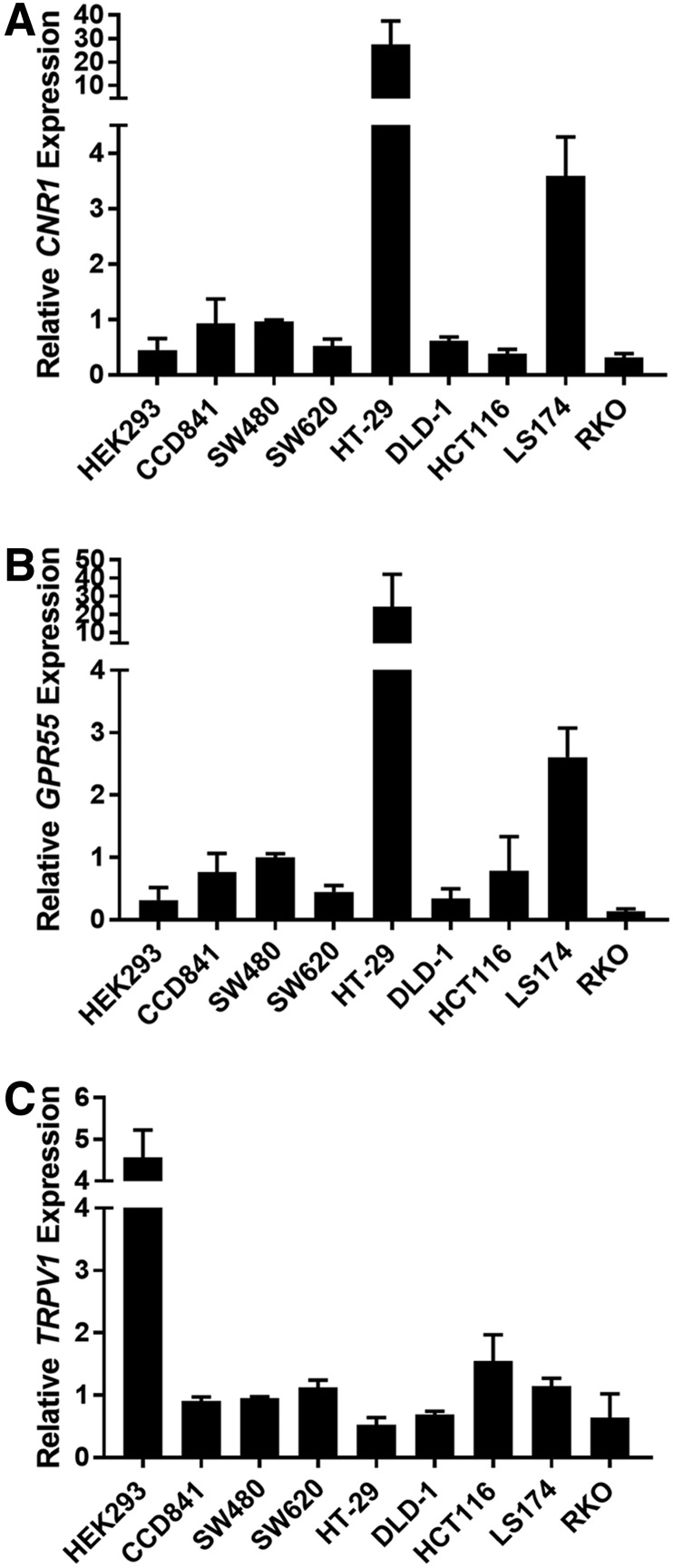
Cannabinoid receptors are expressed in CRC cell lines. RT-qPCR was performed to measure the levels of mRNA for each cannabinoid receptor in the seven CRC cell lines, and the control HEK 293 and CCD 814 CoTr cells **(A)** *CNR1*, **(B)** *GPR55*, and **(C)** *TRPV1.* Data are relative to *GAPDH* mRNA levels and are normalized to the expression in SW480 cells. As noted in the text, *CNR2* RT-qPCR failed to produce reliable amplification curves (due to low or absent mRNA levels). Error bars are SEM. In general, HT-29 and LS174 cells expressed significantly higher levels of *CNR1* and *GPR55* mRNA (*p*≤0.05). CRC, colorectal cancer; mRNA, messenger RNA; RT-qPCR, reverse transcribed-quantitative polymerase chain reaction; SEM, standard error of the mean.

To determine if cannabinoid receptor messenger RNA (mRNA) expression levels vary in our cell line panel (seven colon cancer cell lines, a normal colon epithelial cell line, and HEK 293 cells), we conducted reverse transcribed-quantitative polymerase chain reaction (RT-qPCR) analysis of the four most common cannabinoid receptors (*CNR1*, *CNR2*, *GPR55*, and *TRPV1*). Levels of cannabinoid receptor 1 (*CNR1*) were similar between most of the cell lines tested; however, mRNA expression was markedly higher in HT29 and LS174 cells compared with other cell lines (Fig. 1A). The expression of cannabinoid receptor 2 (*CNR2*) was undetected in each of the cell lines tested (using multiple dilutions of the prepared cDNA). The expression of G protein-coupled receptor 55 (*GPR55*), sometimes referred to as CB3,^21,22^ was similar to that observed for *CNR1*, with the highest expression in HT29 and LS174 cells (Fig. 1B). The expression of transient receptor potential cation channel subfamily V member 1 (*TRPV1*) was similar between all colon cell lines (Fig. 1C).

### Screening of synthetic cannabinoids

**FIG. 2.**
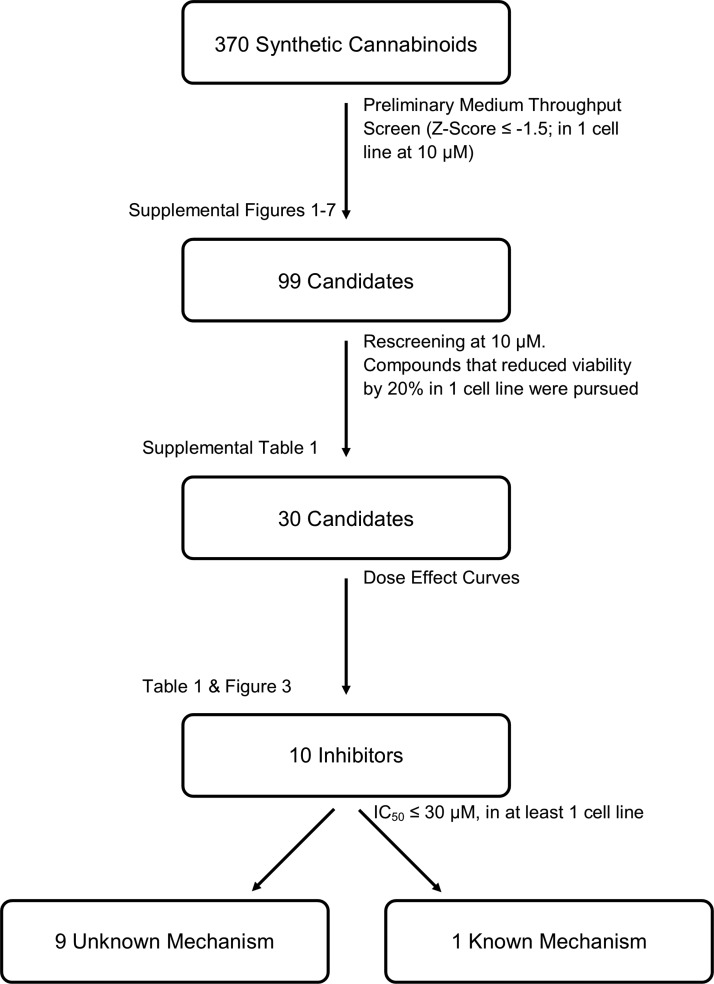
Cannabinoid screening protocol. This schematic illustrates the procedure used for screening the 370 synthetic cannabinoid library against seven CRC cells.

**FIG. 3.**
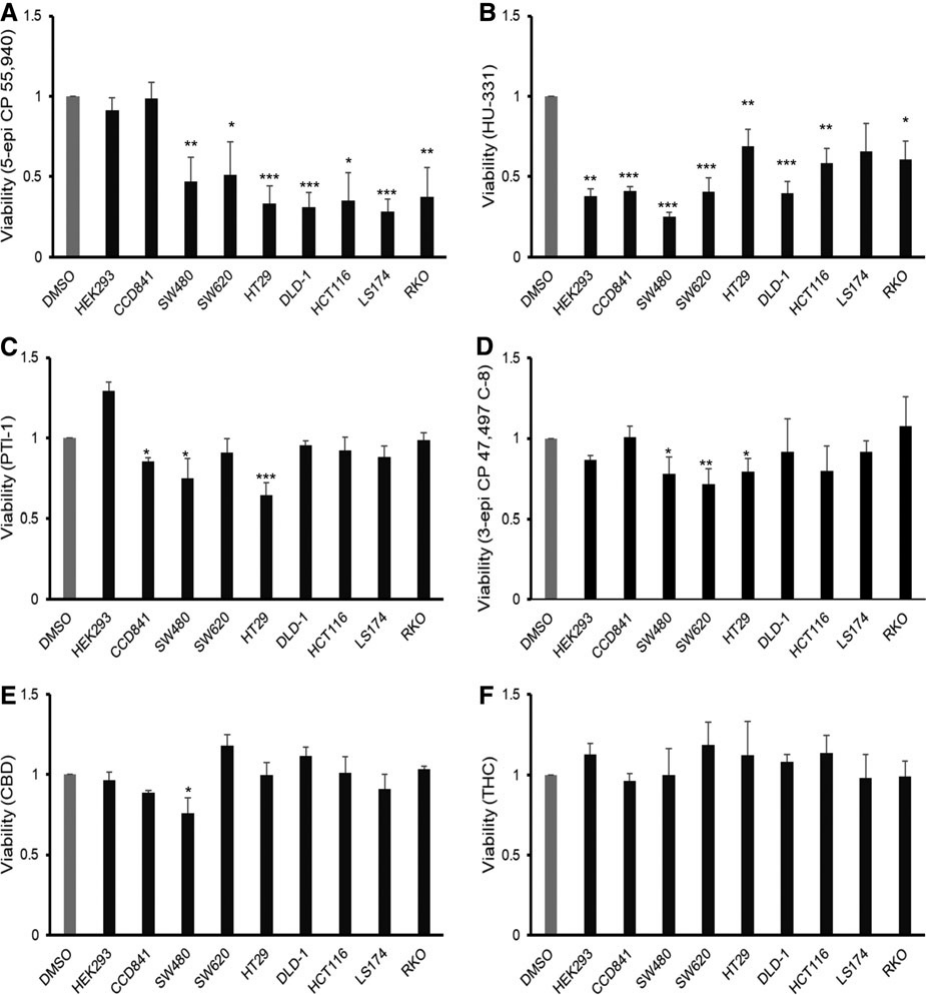
Synthetic cannabinoid compounds reduce the viability of CRC cell lines. Cell viability was assessed with MTS assay 48 h after treatment with selected cannabinoid compounds at a concentration of 10 μM: **(A)** 5-epi CP 55,940, **(B)** HU-331, **(C)** PTI-1, **(D)** 3-epi CP 47,497 C-8 homolog, **(E)** CBD, and **(F)** THC. The DMSO control bar is included as a general reference as viability for each of the cell lines was normalized to its own vehicle (DMSO) treatment (conducted in triplicate). Error bars are SEM; *p≤0.05, **p≤0.01, ***p≤0.001.

While others have reported that cannabinoids can reduce the viability of cancer cell lines, this is the first study to examine a large number of synthetic cannabinoids to reduce CRC cell viability. We screened the ability of 370 synthetic cannabinoids to reduce the viability of seven CRC cell lines, at a screening concentration of 10 μM; the entire screening process is illustrated in Figure 2. Following our screening of the library (Supplementary Figs. S1–S7 [SW480], S2 [SW620], S3 [HT29], S4 [DLD-1], S5 [HCT116], S6 [LS174], and S7 [RKO]), we identified 99 compounds that reduced cell viability, as measured by MTS assay, by at least 1.5 standard deviations, in at least one cell line. To ensure that we did not miss any potentially therapeutic compounds, all of these 99 compounds were subsequently rescreened against all seven CRC cell lines. To minimize false-negative findings, compounds that reduced viability of at least one cell line during original screening and reduced viability of the original cell line or a new cell line during one of the subsequent screenings were pursued further at this point. Of these 99 compounds, we identified 30 compounds that repeatedly reduced cell viability of at least one of the cell lines. Results from representative screenings are shown in Figure 3, and the full list of 30 compounds can be found in Supplementary Table S1. Results of the MTS assay, for select compounds, were verified by trypan blue staining and cell counting (Supplementary Fig. S8).

### Phytocannabinoids have a limited impact on cell growth

The two most abundant phytocannabinoids, THC and CBD, were tested to determine their potential impact on cell growth. Cells were treated at a dose of 10 μM for 48 h and viability was determined with an MTS assay. THC did not impact cell viability against any of the seven lines tested (Fig. 3F). CBD had an effect on SW480 cells and a more modest impact on LS174 cells but did not reduce viability of the other five cell lines tested (Fig. 3E).

### Identification of cannabinoids with anti-CRC potential

**FIG. 4.**
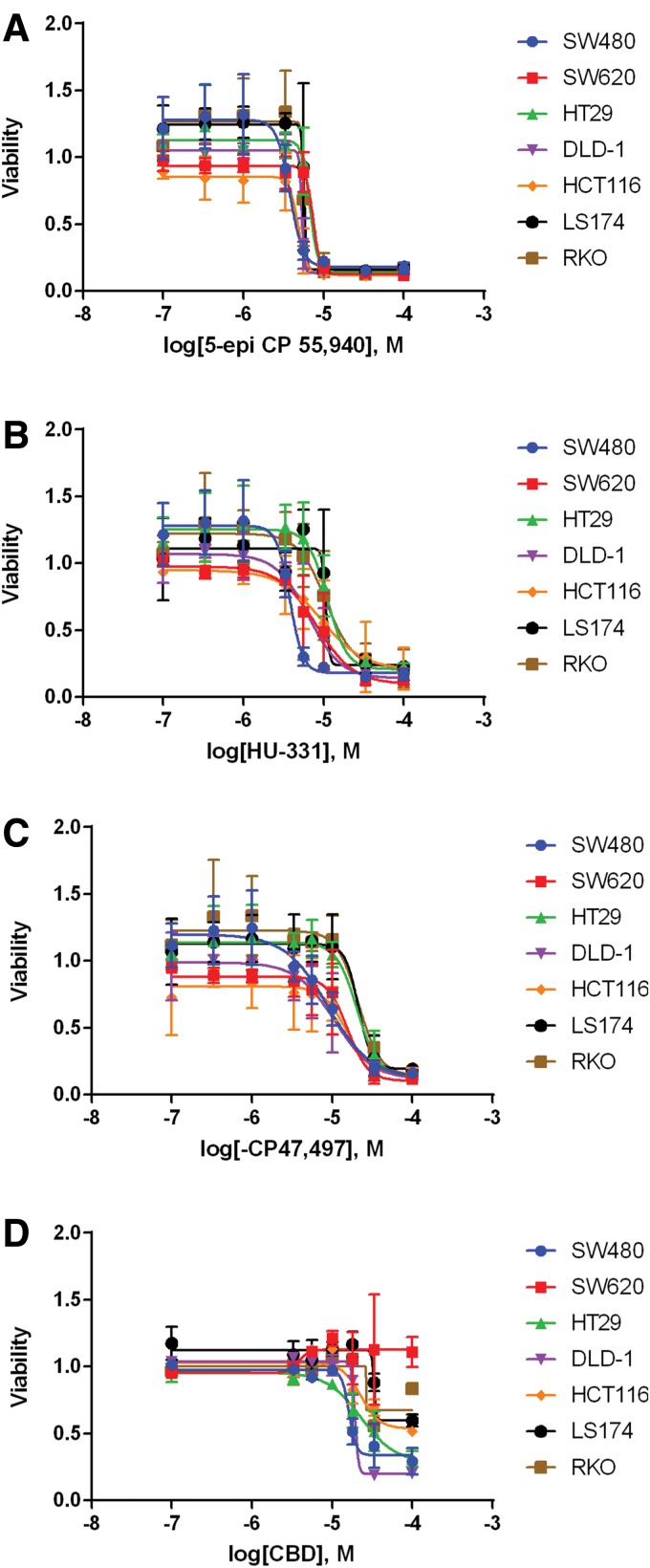
Dose–response curves for selected cannabinoid compounds in CRC cell lines. Cell viability was assessed with MTS assay 48 h after treatment with selected cannabinoid compounds at concentrations of 100 nM, 333 nM, 1 μM, 3.3 μM, 5.6 μM, 10 μM, 33 μM, and 100 μM: **(A)** 5-epi CP 55,940, **(B)** HU-331, **(C)** (−) CP 47,497, and **(D)** CBD. Viability was normalized to vehicle (DMSO)-treated cells. Error bars are SEM.

Dose–response curves were performed for each of the 30 compounds that reduced viability of the rescreening process (we also included CBD and THC as natural phytocannabinoids). Of these compounds, only 10 were identified that had an IC_50_ ≤ 30 μM against one or more CRC cell lines. Sample–dose response curves are shown in Figure 4, and IC_50_ values for the 10 compounds and CBD are presented in Table 1. Importantly, seven of these compounds were selective for reducing the viability of CRC cells as they did not reduce the viability of HEK 293 or CCD 841 CoTr cells. Of the 10 compounds, HU-331 (a known inhibitor of topoisomerase II^23^), PTI-2, and PTI-1 were not selective and inhibited normal epithelial cells and/or HEK 293 cells (data not shown).

**Table 1.** **IC_50_ Values of Identified Cannabinoid Compounds**

Compound	SW480	SW620	HT29	DLD-1	HCT116	LS174	RKO
CBD	16.4±0.6	n.d.	23.0±4.4	19.8±1.4	n.d.	n.d.	n.d.
HU-331	5.5±1.6	11.1±2.5	17.0±3.2	7.8±1.8	11.0±4.2	8.36±2.3	10.4±2.1
(±)-5-epi CP 55,940	6.5±1.6	8.1±1.0	7.3±1.0	5.3±0.04	4.9±0.5	6.2±0.5	5.9±0.5
(±) CP 55,940	25.1±3.1	26.8±2.7	21.3±5.5	21.7±2.6	16.2±5.6	16.3±2.2	14.9±1.7
(+) CP 55,940	24.4±5.6	31.1±3.5	24.1±4.6	16.0±1.2	16.8±4.0	16.9±3.6	19.0±3.3
(−) CP 47,497	8.9±0.1	16.5±6.5	24.6±5.7	12.6±2.3	14.7±0.02	23.0±6.1	19.8±4.4
(±) 3-epi CP 47,497 C-8 Homolog	8.9±1.7	13.5±1.4	14.2±5.0	12.4±2.0	12.6±1.5	12.2±0.9	15.0±2.8
(±) CP 47,497 C-8 Homolog	n.d.	n.d.	20.1±4.2	33.4±1.8	32.0±1.8	21.7±6.1	39.0±5.6
PTI-1	11.9±2.3	19.6±0.2	14.4±2.4	19.4±1.1	21.2±5.5	25.0±3.6	27.5±2.7
PTI-2	7.4±1.4	23.9±3.6	8.2±1.6	34.2±7.8	27.7±3.1	n.d.	15.6±2.9
NPB-22	9.7±0.6	n.d.	n.d.	n.d.	15.2±6.3	n.d.	n.d.

Concentrations are in μM, and “n.d.” indicates that an IC_50_ could not be determined from the dose–response curve (because either there was no reduction in cell viability or cell viability failed to fall below 50%). IC_50_ values varied from 4.9 to 39 μM, and there were no statistically significant differences within this large dataset.

THC, while not effective at reducing cell viability at 10 μM, did reduce cell viability at higher concentrations. THC was effective in SW480 (Supplementary Fig. S9A) and HCT116 (Supplementary Fig. S9B) cells, but did not reduce viability below 50% even at the highest dose tested (100 μM). CBD, however, was effective at reducing cell viability of SW480 cells below 50% (Supplementary Fig. S9A), but was only slightly more potent than THC in HCT116 cells (Supplementary Fig. S9B).

### Identified cannabinoids did not utilize canonical pathways

**FIG. 5.**
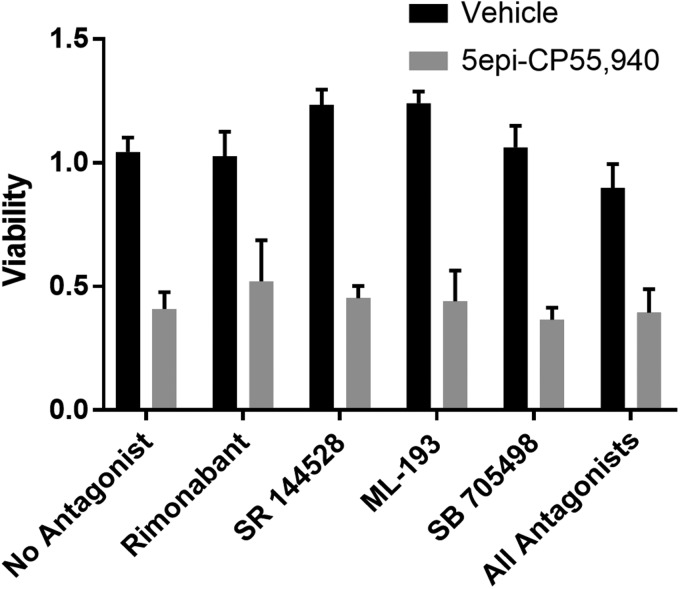
Antagonists to canonical cannabinoid receptors do not prevent the reduction of cell viability. Cell viability was assessed in SW480 cells 48 h after treatment with 5-epi CP 55,940 at a concentration of 5 μM in the presence of vehicle, rimonabant (CB1 antagonist), SR 144528 (CB2 antagonist), ML-193 (GPR55 antagonist), SB-705498 (TRPV1 antagonist), or all four antagonists at 10 μM. Viability was normalized to vehicle (DMSO)-treated cells in four standard wells on each plate. Error bars are SEM. There were no statistically significant differences between 5-epi CP 55,940-treated groups, co-treated with either antagonist or vehicle. However, all of the 5-epi CP 55,940-treated groups displayed significantly reduced viability compared with their cognate vehicle controls (*p*≤0.05). SR 144528 and ML-193 modestly, but significantly, increased viability of SW480 cells (*p*≤0.05).

We next sought to identify which cannabinoid receptor was mediating the action of our identified cannabinoids. We repeated the cannabinoid treatment with our most potent cannabinoid [(±)-5-epi CP 55,940] and antagonists to each of the four major receptors using SW480 cells. Treatment with these antagonists (alone or in combination) failed to prevent the reduction in cell viability observed with (±)-5-epi CP 55,940 (Fig. 5).

## Discussion

**FIG. 6.**
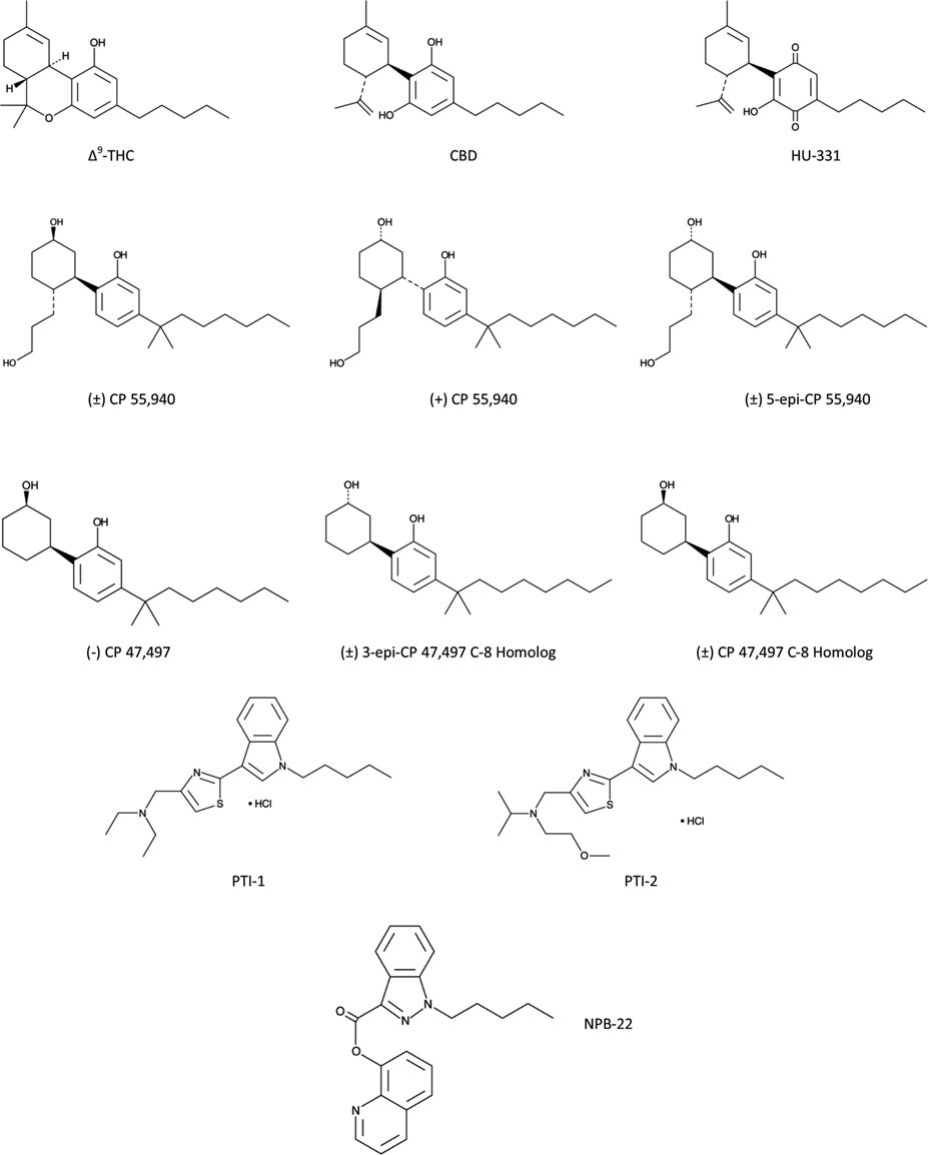
Structure of compounds that reduced viability of CRC cell lines below 50%. Shown are the structures of 10 synthetic cannabinoid compounds (compared with CBD and THC) that were shown to reproducibly reduce cell viability of CRC cells.

Previous work has demonstrated the ability of cannabinoid compounds to reduce the viability of CRC cell lines.^8,9,24–28^ However, a large-scale screening of these compounds to assess the potency and efficacies of different synthetic cannabinoids has not previously been performed. Here, we demonstrated that 10 synthetic compounds are highly efficacious and moderately potent for reducing the viability of seven CRC cell lines. The structures of these compounds are illustrated in Figure 6. In terms of rigor and reproducibility, we sought compounds that displayed activity against seven different cell lines derived from independent sources. Interestingly, all seven lines each express mRNA for three of the four accepted cannabinoid receptors (CB1, GPR55, and TRPV1, but not CB2), although at different levels. Of the 10 compounds, 7 appear to be selective for CRC cells as these compounds did not reduce the viability of HEK 293 or CCD 841 CoTr cells. Only HU-331 and PTI-2 reduced viability of all of the cell lines tested, including HEK 293 and CCD 841 CoTr cells; PTI-1 also reduced viability of CCD 841 CoTr Cells.

Interestingly, these compounds can be placed into three families: CP 55,940, CP 47,497, and PTI. Additionally, both CBD and the synthetic derivative of CBD, HU-331, exhibited the ability to reduce CRC cell viability, although CBD displayed a weak activity with an IC_50_ of 16.3–23.1 μM and efficacy in only three cell lines. Additionally, for CP 55,940 and CP 47,497, we found that certain isomers were more potent than others. In particular, of the four CP 55,940 compounds in the library, only one [(−)-CP 55,940] failed to reduce CRC cell viability, suggesting that the (+)-stereoisomer is an active form as this was present in the other three derivatives. With regard to CP 47,497, the library contains nine derivatives of which only three reduced viability by >50% (one additional compound reduced viability between 20% and 25% in some cell lines). Of the three compounds that were identified, two were stereoisomers of the more potent C-8 homolog and one was a stereoisomer of the parental C-7 isomer, (−)-CP 47,497.^29^ Mixtures containing the (+)-stereoisomer of the parental compound or the (+)-stereoisomer alone were unable to reduce CRC cell viability. Additionally, neither the para-quinone analog nor the C-6 or C-9 homologs were able to reduce CRC cell viability.

Despite having a similar binding affinity for CB1 and CB2 as CP 55,940 and CP 47,497, WIN 55,212-2 was much less potent and less efficacious than these two compounds (data not shown; Supplementary Table S1). One explanation for this is that CP 55,940 has been found to act as an antagonist against GPR55, while WIN 55,212-2 has no appreciable activity at this receptor.^30–32^ The ability of CP 55,940 to antagonize GPR55 is consistent with our finding that CBD, but not THC, was able to reduce the viability of CRC cells. THC is an agonist of GPR55, whereas CBD has been shown to be an antagonist of GPR55.^33^ There are no published data on the pharmacological activity of the identified synthetic cannabinoids against GPR55. While this is an attractive idea, we did not detect any effect on cell viability by GPR55 antagonist ML-193. This suggests that the cannabinoids may be signaling through GPR55 in a manner different from ML-193, or that antagonism of GPR55 is not the mechanism of action of the identified cannabinoids.

It is unclear from our study which receptor is responsible for reducing cell viability by these 11 cannabinoids. Studies using receptor antagonists of CB1, CB2, GPR55, and TRPV1 failed to block the action of 5-epi CP 55,940 on SW480 cells (Fig. 5). This lack of blockade by antagonists was true even when all four antagonists were combined (Fig. 5). This suggests that 5-epi CP 55,940 is likely acting through a noncanonical mechanism. Additional studies will be needed to identify potential receptors/signaling pathways that mediate this response.

We also noted a marked difference in the potency and efficacies of these compounds in the various cell lines tested. Some compounds, such as 5-epi CP 55,940, were both potent and efficacious in all seven cell lines, while others such as NPB-22 were only efficacious in two cell lines. This was likely due to individual mutations within the specific cell lines. This was supported by the observation that the (±)-CP 47,497 C-8 homolog while of low potency was efficacious in five of the cell lines tested, but was ineffective in both SW480 and SW620 cells that were derived from the same patient. We also failed to observe a complete reduction of viability of any cell line. Our most potent and efficacious compounds only reduced viability to between 10% and 15% even at the highest dose (100 μM) tested. This could be due to new cell divisions occurring during the 48 h treatment period, and the daughter cells not being exposed to the synthetic cannabinoid for a long enough time period. This finding could also be due to the degradation of cannabinoid or reduced concentration of the cannabinoid over time. It is also possible that, given the limited solubility of these agents, we were simply unable to deliver high enough concentrations.

In the majority of CRC tumors, there is aberrant activation of the Wnt/β-catenin pathway. Of the cell lines tested, six of the seven had mutations that activated this pathway; however, we did not observe any difference in susceptibility to cannabinoids based on the activation of this pathway. Interestingly, we did find that only tumor cell lines isolated from primary tumors, with mutations in the APC gene (SW480, HT29, and DLD-1), were more susceptible to CBD, and cells that contained mutations in the β-catenin gene (HCT116 and LS174) were only moderately impacted by CBD where viability did not typically drop below 50% even at the highest concentrations tested. The treatment-resistant metastatic cell line SW620 was also not susceptible to CBD. This might suggest that CBD is able to reduce the activity of Wnt/β-catenin pathway before the translocation of β-catenin into the nucleus.

In summary, we identified 10 synthetic cannabinoids demonstrating reproducible activity against human cells, with seven compounds reducing viability only in CRC cells. These compounds fall into three families of molecules, CP 55,940, CP 47,497, and PTI. Interestingly, CBD had only incomplete activity against a subset of CRC lines, and THC was without activity at 10 μM. We also identified potential isomers and modifications that may contribute to increasing the potency and efficacy of CP 55,940 and CP 47,497 that may lead to novel drugs to treat this deadly disease.

## Supplementary Material

Supplemental data

Supplemental data

Supplemental data

Supplemental data

## Abbreviations Used

CBD: cannabidiol
CRC: colorectal cancer
mRNA: messenger RNA
SEM: standard error of the mean
THC: Δ^9^-tetrahydrocannabinol
