# Supplementary material for: Synthetic Cannabinoid Activity Against Colorectal Cancer Cells
Source: Cannabis Cannabinoid Res. 2018 Dec 21;3(1):272–81. doi: 10.1089/can.2018.0065 (PMC6340378; doi:10.1089/can.2018.0065)
Supplement: Supplemental data [file Supp_Figs1-7.pdf]

Supplementary Data

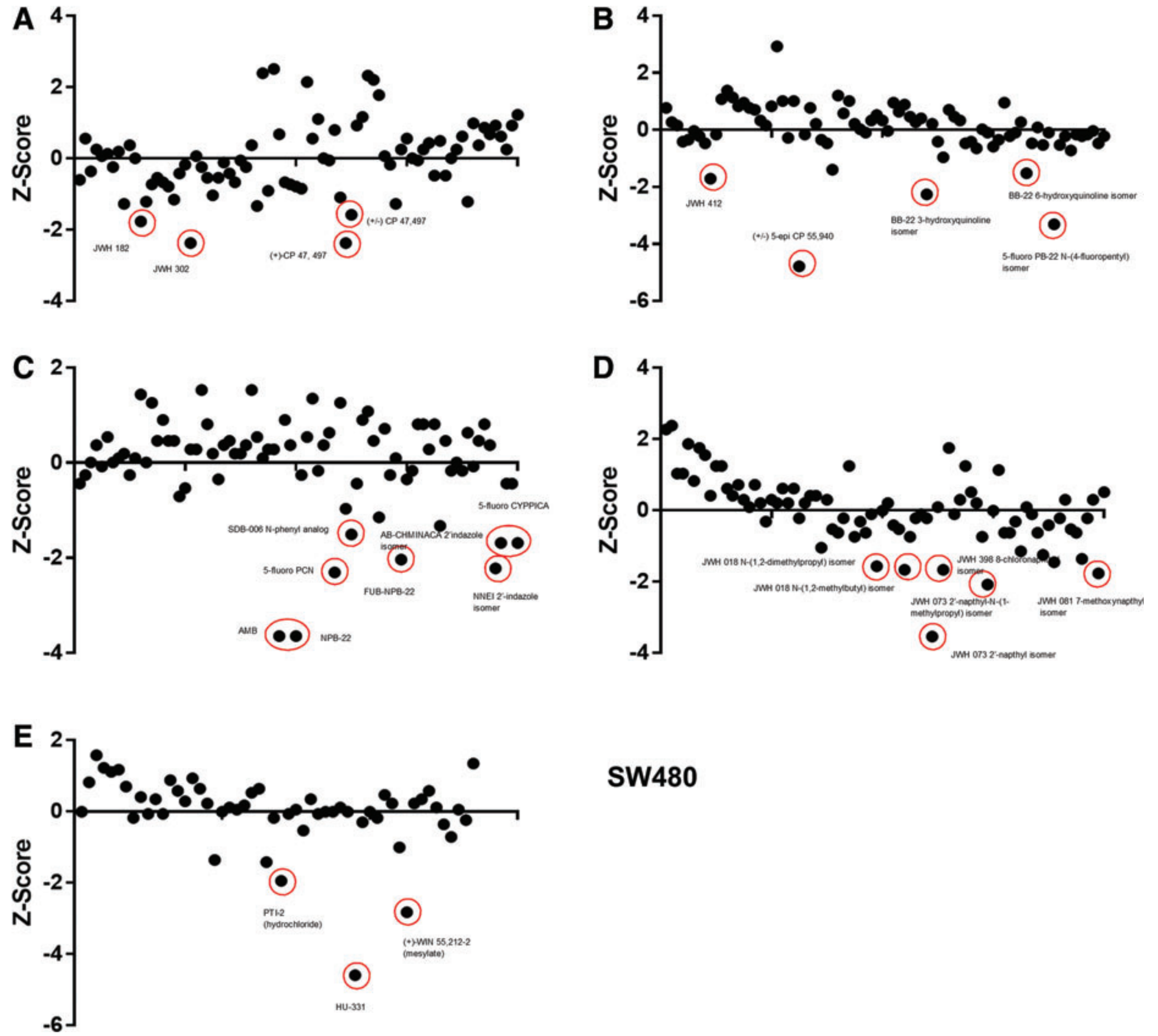

SUPPLEMENTARY FIG. S1. Library screening of SW480 colorectal cancer cell lines. To demonstrate assay performance characteristics, results from the primary screens in the SW480 colorectal cancer cell lines are provided. Cells were treated with cannabinoids from the synthetic library at 10 μM for 48 h, and cell viability was measured with MTS assay. Plots of z-scores in SW480 cells are shown for each library plate: **(A)** plate 1, **(B)** plate 2, **(C)** plate 3, **(D)** plate 4, **(E)** plate 5; red circles indicate compounds that had z-scores ≤ -1.5 and were rescreened.

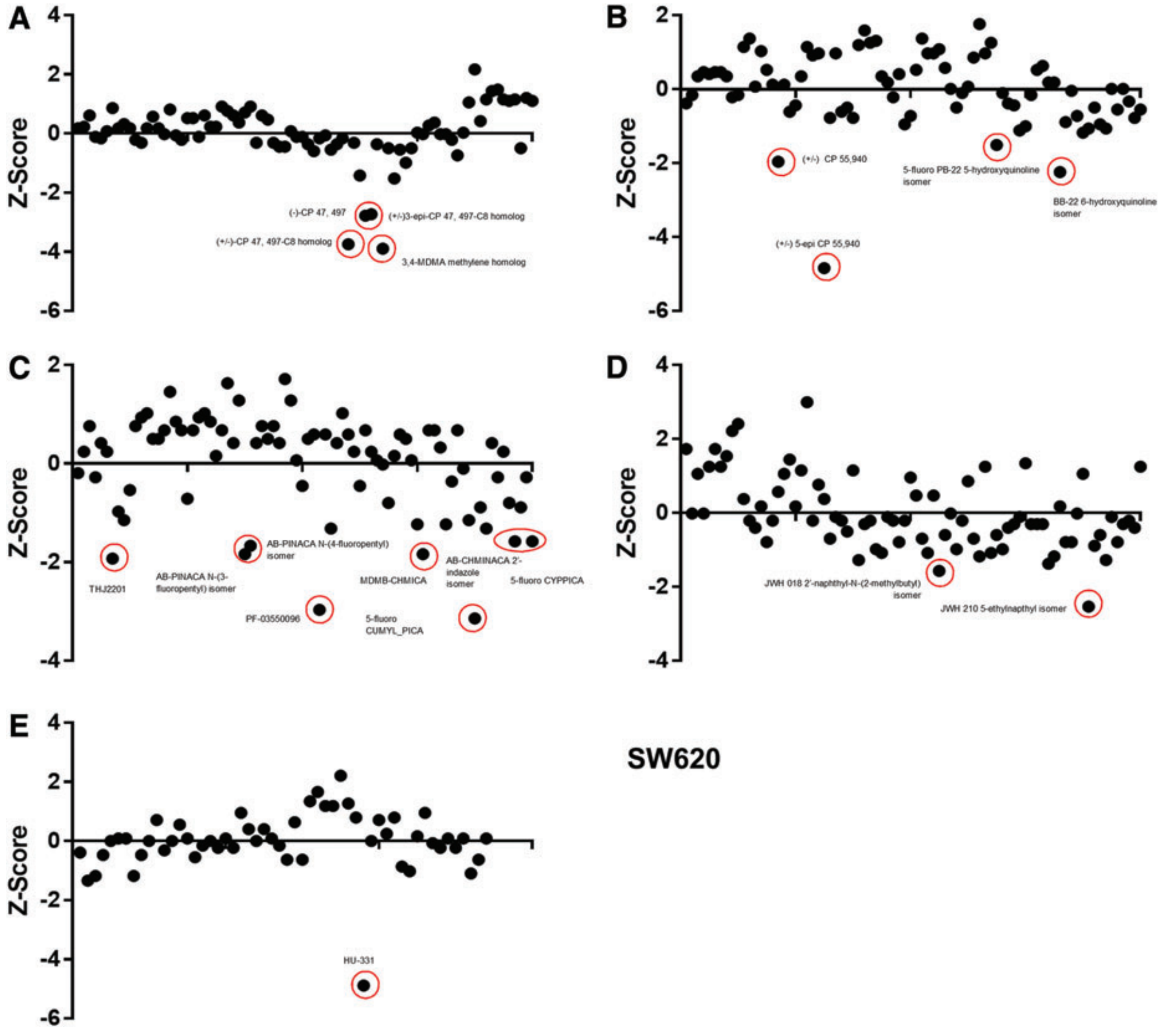

SUPPLEMENTARY FIG. S2. Library screening of SW620 colorectal cancer cell lines. To demonstrate assay performance characteristics, results from the primary screens in the SW620 colorectal cancer cell lines are provided. Cells were treated with cannabinoids from the synthetic library at 10 μM for 48 h, and cell viability was measured with MTS assay. Plots of z-scores in SW480 cells are shown for each library plate: **(A)** plate 1, **(B)** plate 2, **(C)** plate 3, **(D)** plate 4, **(E)** plate 5; red circles indicate compounds that had z-scores ≤ -1.5 and were rescreened.

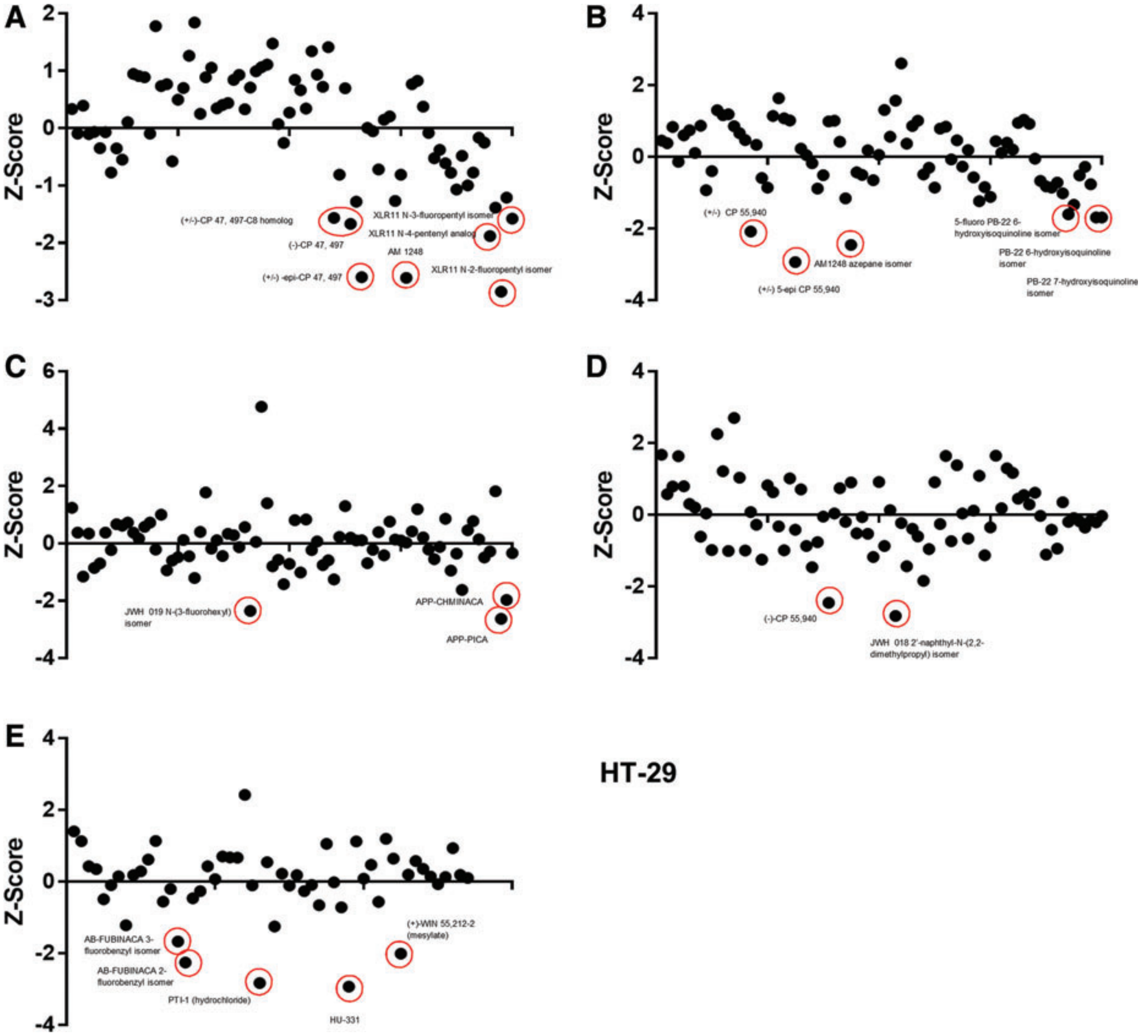

SUPPLEMENTARY FIG. S3. Library screening of HT29 colorectal cancer cell lines. To demonstrate assay performance characteristics, results from the primary screens in the HT29 colorectal cancer cell lines are provided. Cells were treated with cannabinoids from the synthetic library at 10 μM for 48 h, and cell viability was measured with MTS assay. Plots of z-scores in SW480 cells are shown for each library plate: **(A)** plate 1, **(B)** plate 2, **(C)** plate 3, **(D)** plate 4, **(E)** plate 5; red circles indicate compounds that had z-scores ≤ -1.5 and were rescreened.

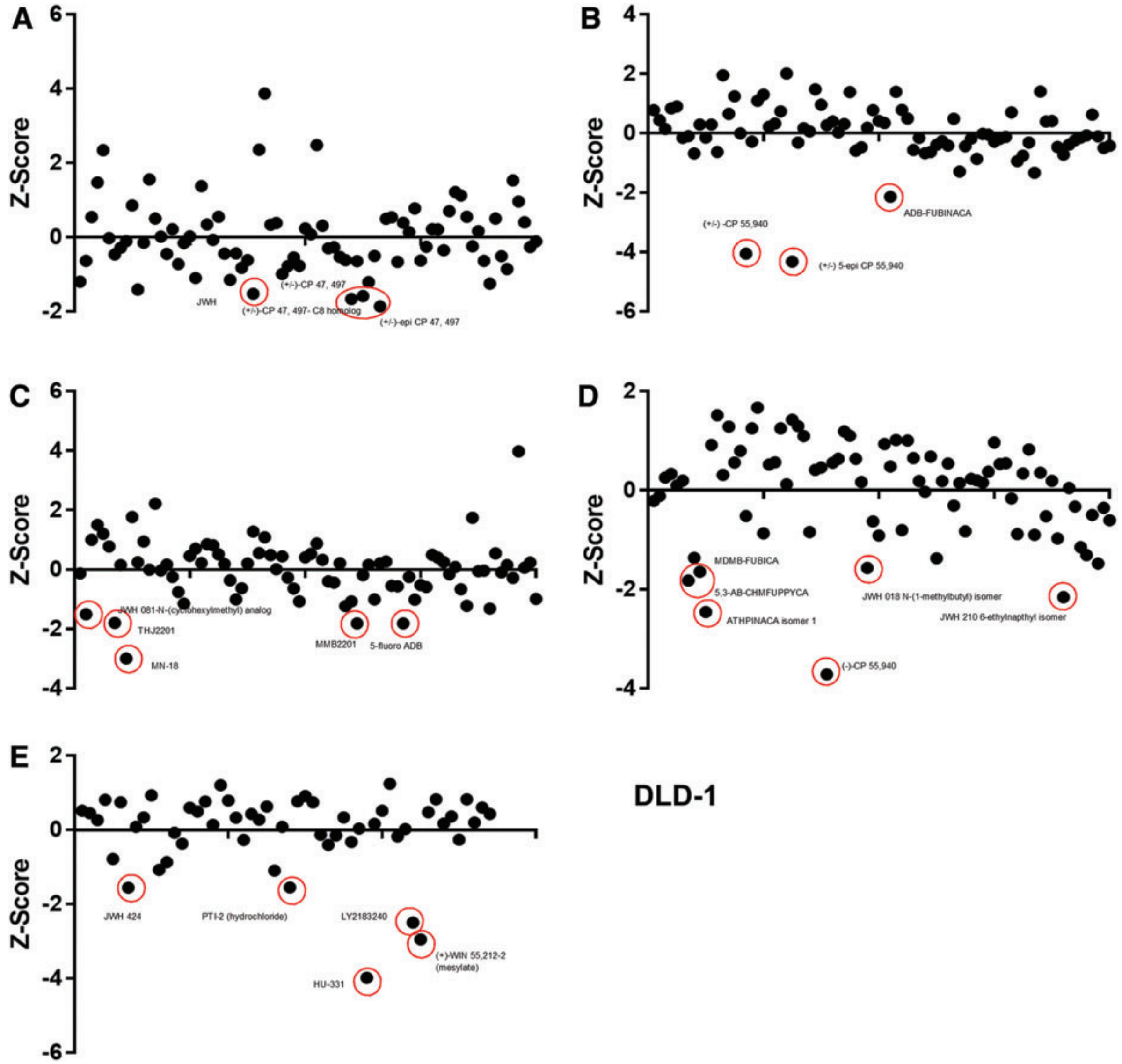

SUPPLEMENTARY FIG. S4. Library screening of DLD-1 colorectal cancer cell lines. To demonstrate assay performance characteristics, results from the primary screens in the DLD-1 colorectal cancer cell lines are provided. Cells were treated with cannabinoids from the synthetic library at 10 μM for 48 h, and cell viability was measured with MTS assay. Plots of z-scores in SW480 cells are shown for each library plate: **(A)** plate 1, **(B)** plate 2, **(C)** plate 3, **(D)** plate 4, **(E)** plate 5; red circles indicate compounds that had z-scores ≤ -1.5 and were rescreened.

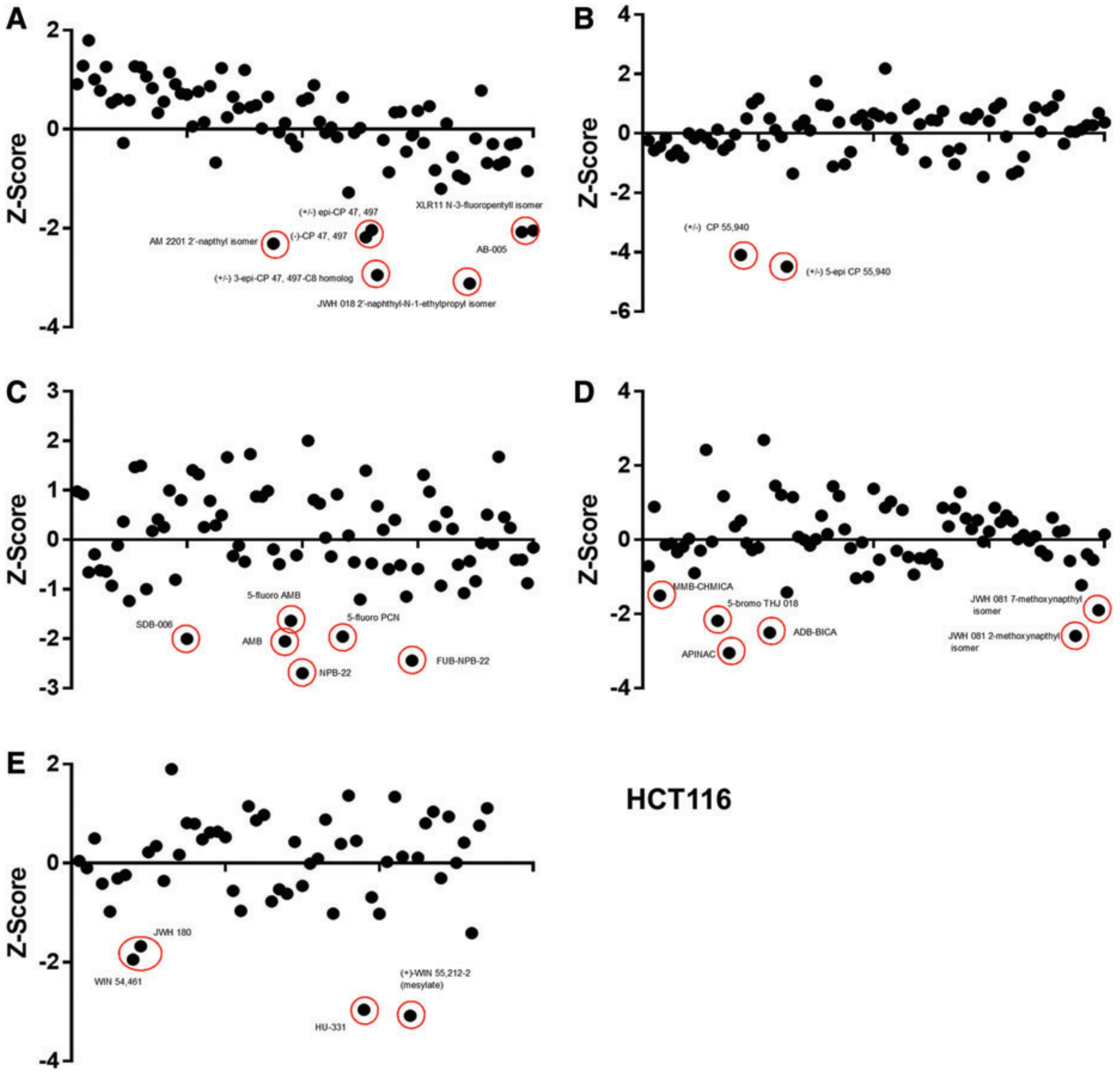

SUPPLEMENTARY FIG. S5. Library screening of HCT116 colorectal cancer cell lines. To demonstrate assay performance characteristics, results from the primary screens in the HCT116 colorectal cancer cell lines are provided. Cells were treated with cannabinoids from the synthetic library at 10 μM for 48 h, and cell viability was measured by MTS assay. Plots of z-scores in SW480 cells are shown for each library plate: **(A)** plate 1, **(B)** plate 2, **(C)** plate 3, **(D)** plate 4, **(E)** plate 5; red circles indicate compounds that had z-scores ≤ -1.5 and were rescreened.

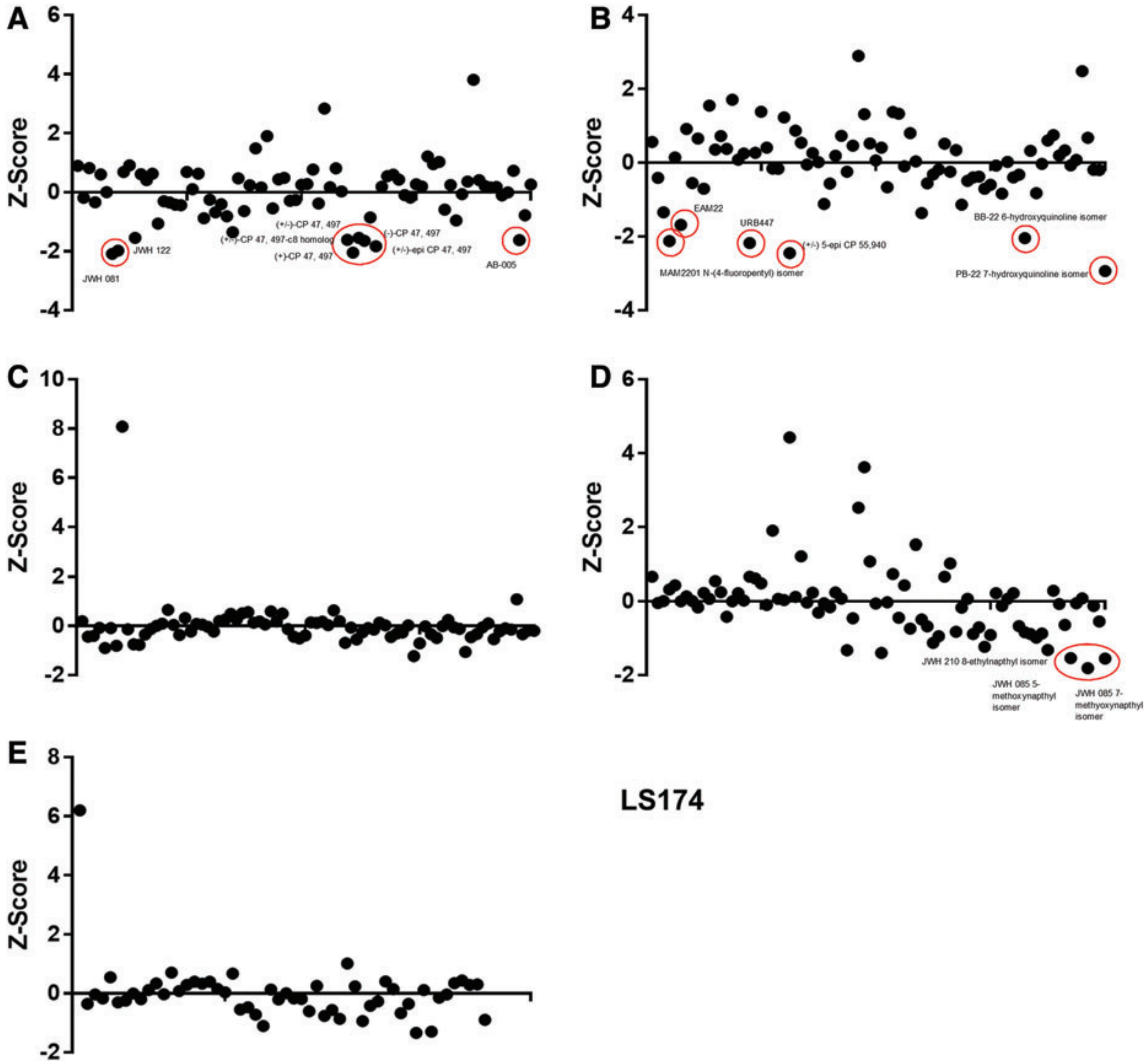

SUPPLEMENTARY FIG. S6. Library screening of LS174 colorectal cancer cell lines. To demonstrate assay performance characteristics, results from the primary screens in the LS174 colorectal cancer cell lines are provided. Cells were treated with cannabinoids from the synthetic library at 10 μM for 48 h, and cell viability was measured by MTS assay. Plots of z-scores in SW480 cells are shown for each library plate: **(A)** plate 1, **(B)** plate 2, **(C)** plate 3, **(D)** plate 4, **(E)** plate 5; red circles indicate compounds that had z-scores ≤ -1.5 and were rescreened.

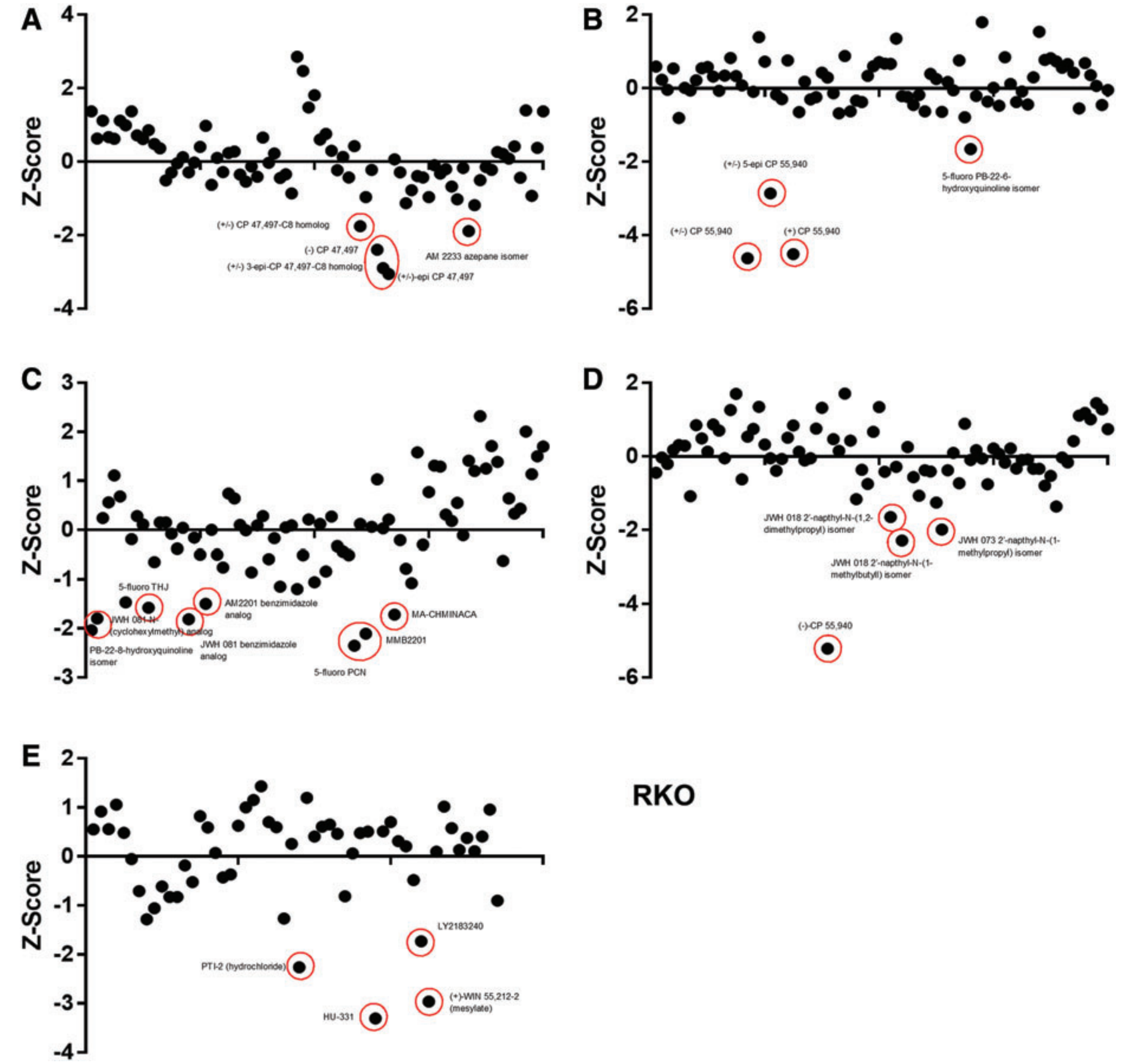

SUPPLEMENTARY FIG. S7. Library screening of RKO colorectal cancer cell lines. To demonstrate assay performance characteristics, results from the primary screens in the RKO colorectal cancer cell lines are provided. Cells were treated with cannabinoids from the synthetic library at 10 μM for 48 h, and cell viability was measured by MTS assay. Plots of z-scores in SW480 cells are shown for each library plate: **(A)** plate 1, **(B)** plate 2, **(C)** plate 3, **(D)** plate 4, **(E)** plate 5; red circles indicate compounds that had z-scores ≤ -1.5 and were rescreened.
