# Supplementary material for: Synthetic Cannabinoid Activity Against Colorectal Cancer Cells
Source: Cannabis Cannabinoid Res. 2018 Dec 21;3(1):272–81. doi: 10.1089/can.2018.0065 (PMC6340378; doi:10.1089/can.2018.0065)
Supplement: Supplemental data [file Supp_Table1.pdf]

Supplementary Table S1. Cannabinoid Compounds That Were Found to Reduce Colorectal Cancer Cell Viability

Compound	SW480	SW620	HT29	DLD-1	HCT116	LS174	RKO
DMSO	110.5 ± 4.6	110.8 ± 4.8	111.0 ± 4.5	113.1 ± 7.2	101.3 ± 11.8	99.3 ± 5.1	110.0 ± 8.7
CBD	75.8 ± 9.9*	118.1 ± 6.7	99.5 ± 8.1	111.4 ± 5.5	100.9 ± 10.0	91.1 ± 9.0	103.6 ± 1.7
THC	100.0 ± 16.5	118.5 ± 14.2	112.0 ± 21.3	108.3 ± 4.5	113.5 ± 11.1	98.1 ± 14.6	99.0 ± 9.7
JWH 018 benzimidazole analog	91.5 ± 18.2	90.1 ± 12.3	108.7 ± 8.0	109.6 ± 5.4	88.3 ± 7.5	100.5 ± 10.8	94.9 ± 6.8
PTI-2 (hydrochloride)	62.6 ± 8.9**	80.6 ± 6.9**	77.0 ± 12.1*	95.0 ± 6.0	97.3 ± 9.6	89.4 ± 10.8	86.8 ± 100
(+)-WIN 55,212-2 (mesylate)	68.2 ± 9.5**	76.5 ± 11.1*	75.5 ± 1.2*	78.8 ± 10.6*	103.0 ± 10.0	78.5 ± 12.5	80.6 ± 12.4
HU-331	25.1 ± 2.5***	40.8 ± 8.3***	69.0 ± 10.7**	40.7 ± 6.6***	45.9 ± 10.1**	69.0 ± 16.6	68.9 ± 9.8*
(±)5-epi CP 55,940	47.1 ± 15.1**	51.0 ± 20.7*	33.2 ± 11.0***	30.8 ± 9.1***	35.0 ± 17.3*	28.2 ± 7.6***	37.4 ± 7.8**
(±)-CP 55,940	120.9 ± 81	108.0 ± 10.2	99.7 ± 12.6	87.2 ± 14.2	72.2 ± 14.4	117.2 ± 5.0	107.0 ± 21.1
(±)-CP 47,497-C8 homolog	111.1 ± 12.1	93.2 ± 16.1	104.1 ± 9.6	100.7 ± 8.3	86.4 ± 10.4	102.8 ± 7.9	107.2 ± 9.6
(–)-CP 47,497	81.6 ± 8.4*	78.5 ± 12.6	90.8 ± 9.9	80.9 ± 12.1	78.8 ± 9.3	98.1 ± 9.3	101.3 ± 10.8
(±)-3-epi-CP 47,497-C8-homolog	77.8 ± 10.4*	71.5 ± 9.7**	79.2 ± 8.3*	91.7 ± 20.5	88.0 ± 9.6	91.8 ± 6.5	107.7 ± 18.0
(±)-epi CP 47,497	124.1 ± 11.3	99.8 ± 6.6	101.6 ± 8.8	98.6 ± 4.9	86.1 ± 7.5	96.9 ± 10.7	103.1 ± 16.8
XLR11 N-4-pentenyl analog	125.4 ± 13.2	117.8 ± 5.0	102.7 ± 12.4	112.3 ± 8.5	114.8 ± 8.2	103.7 ± 8.2	102.2 ± 7.5
AM 2233 azepane isomer	108.3 ± 17.4	104.1 ± 5.5	116.2 ± 6.7	116.9 ± 5.7	117.3 ± 13.7	104.1 ± 58	111.4 ± 11.8
AM1248 azepane Isomer	125.1 ± 10.4	119.2 ± 11.3	95.6 ± 15.1	115.3 ± 68	107.1 ± 10.2	112.1 ± 5.1	121.1 ± 11.4
(+)-CP 55,940	124.0 ± 10.2	107.5 ± 4.6	114.0 ± 12.6	102.6 ± 3.3	74.2 ± 8.3	107.0 ± 5.8	97.5 ± 11.3
5-fluoro PCN	101.2 ± 4.1	88.8 ± 10.6	106.5 ± 8.8	103.7 ± 5.0	86.8 ± 7.5	86.3 ± 9.2	90.6 ± 6.6
SDB-006 N-phenyl analog	117.1 ± 11.1	96.7 ± 6.0	106.2 ± 12.2	96.8 ± 8.4	92.8 ± 7.1	95.4 ± 14.3	95.8 ± 3.8
MDMB-FUBICA	116.8 ± 15.3	97.9 ± 7.8	105.0 ± 16.8	97.3 ± 6.5	97.6 ± 8.6	105.3 ± 6.8	98.6 ± 9.7
JWH 018 N-(1-methylbutyl) isomer	113.3 ± 11.6	85.5 ± 7.9	96.9 ± 14.2	99.1 ± 7.9	105.5 ± 6.4	103.4 ± 16.0	96.2 ± 7.1
ATHPINACA isomer 1	86.7 ± 5.9*	100.6 ± 4.6	93.9 ± 9.9	87.7 ± 4.9*	84.2 ± 6.8	72.9 ± 9.5	98.2 ± 10.1
LY2183240	110.4 ± 9.8	99.1 ± 5.6	106.1 ± 5.1	96.8 ± 7.9	87.2 ± 12.1	94.2 ± 8.4	84.3 ± 7.4
PB-22 7-hydroxyisoquinoline isomer	109.9 ± 3.9	105.1 ± 3.2	103.6 ± 10.9	100.4 ± 6.5	106.9 ± 6.9	94.0 ± 11.5	109.2 ± 3.9
JWH 398 8-chloronaphthyl isomer	109.7 ± 9.8	97.3 ± 7.7	100.0 ± 7.7	107.0 ± 7.5	99.0 ± 5.4	85.9 ± 8.7	93.2 ± 7.2
AB-005	108.2 ± 5.6	114.9 ± 8.9	103.4 ± 9.9	114.2 ± 4.9	107.9 ± 10.7	95.9 ± 8.8	110.5 ± 13.6
AM1220	107.7 ± 4.0	101.6 ± 2.7	108.1 ± 4.4	103.3 ± 4.0	106.5 ± 2.9	91.4 ± 7.4	104.8 ± 6.6
ADB-FUBINACA	116.8 ± 4.7	111.5 ± 4.9	105.4 ± 11.2	96.3 ± 9.9	114.1 ± 5.0	104.2 ± 5.8	102.5 ± 6.6
BB-22 6-hydroxyisoquinoline isomer	97.1 ± 5.8	98.4 ± 6.3	118.3 ± 8.3	102.1 ± 5.2	87.4 ± 5.1	89.4 ± 6.7	91.2 ± 10.4
NPB-22	68.9 ± 11.3*	101.1 ± 7.4	113.6 ± 6.8	100.1 ± 12.3	73.0 ± 14.7	109.6 ± 10.4	109.6 ± 10.4
PTI-1 (hydrochloride)	74.7 ± 12.6*	91.1 ± 8.7	64.5 ± 7.5***	95.6 ± 2.7	92.3 ± 7.9	88.1 ± 6.8	98.6 ± 4.8
apthyl isomer	93.0 ± 5.8*	92.7 ± 6.9	104.4 ± 6.1	99.4 ± 7.6	91.5 ± 7.1	85.6 ± 5.6	93.2 ± 9.7
MAM2201 N-(4-fluoropentyl) isomer	105.2 ± 5.3	93.6 ± 12.0	108.1 ± 6.8	103.1 ± 2.7	91.8 ± 4.8	101.7 ± 8.3	100.1 ± 2.7

Viability data for the 30 compounds that were identified from our initial and rescreening process at 10 μM after 48 h. It should be noted that compounds were pursued if viability was reduced during two of the three screens in one or more cell lines. Average percentage viability is shown ± standard error of the mean.

Asterisks denote significant differences in percentage viability between treated and vehicle control cells; $*p \leq 0.05$, $**p \leq 0.01$, $***p \leq 0.001$.
