# Supplementary material for: Synthetic Cannabinoid Activity Against Colorectal Cancer Cells
Source: Cannabis Cannabinoid Res. 2018 Dec 21;3(1):272–81. doi: 10.1089/can.2018.0065 (PMC6340378; doi:10.1089/can.2018.0065)
Supplement: Supplemental data [file Supp_Fig8.pdf]

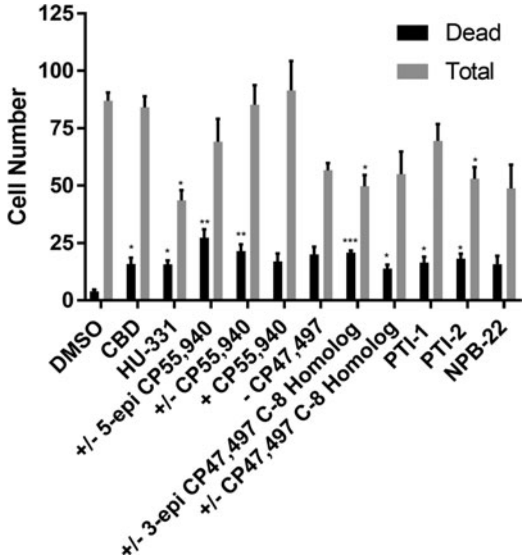

SUPPLEMENTARY FIG. S8. Synthetic cannabinoid compounds reduce proliferation and kill colorectal cancer cells. Cell viability was assessed by cell counting of trypan blue-stained cells 48 h after treatment with selected cannabinoid compounds at a concentration of 10 μM. Cell numbers for dead and total from one hemocytometer grid are shown. Error bars are SEM. Asterisks denote significant differences between treated and vehicle control cell numbers; *$p \leq 0.05$, **$p \leq 0.01$, ***$p \leq 0.001$. SEM, standard error of the mean.
