# Supplementary material for: Synthetic Cannabinoid Activity Against Colorectal Cancer Cells
Source: Cannabis Cannabinoid Res. 2018 Dec 21;3(1):272–81. doi: 10.1089/can.2018.0065 (PMC6340378; doi:10.1089/can.2018.0065)
Supplement: Supplemental data [file Supp_Fig9.pdf]

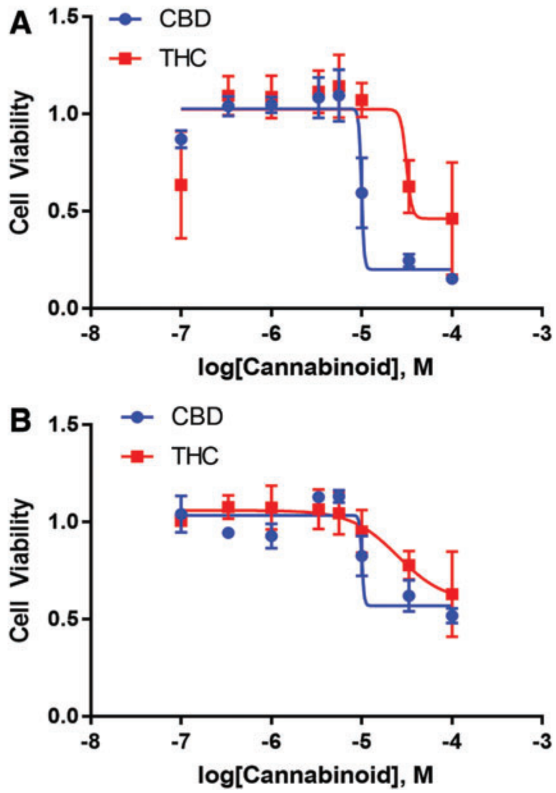

SUPPLEMENTARY FIG. S9. Comparison of THC and CBD potency and efficacy. After treatment with either THC or CBD for 48 h at concentrations of 100 nM, 333 nM, 1 μM, 3.3 μM, 5.6 μM, 10 μM, 33 μM, and 100 μM, cell viability was assessed with MTS assay: **(A)** SW480 cells and **(B)** HCT116 cells. Error bars are standard error of the mean (SEM).
